# Hyper non-CG methylation of expanded plant disease resistance *NLR* genes

**DOI:** 10.1007/s00299-023-03018-5

**Published:** 2023-04-13

**Authors:** Qianqian Cao, Shichao Wang, Huizhen Ma, Sha Luo, Hai He, Yu Zhang

**Affiliations:** 1 School of Agriculture, Sun Yat-Sen University, Shenzhen, 518107 China; 2 Shenzhen Branch, Guangdong Laboratory for Lingnan Modern Agriculture, Genome Analysis Laboratory of the Ministry of Agriculture, Agricultural Genomics Institute at Shenzhen, Chinese Academy of Agricultural Sciences, Shenzhen, 518124 China; 3 State Key Laboratory of Crop Stress Adaptation and Improvement, School of Life Sciences, Henan University, Kaifeng, 475004 China; 4 Shenzhen Research Institute of Henan University, Shenzhen, 518000 China; 5 Department of Horticulture, College of Agronomy, Jiangxi Agricultural University, Nanchang, 330045 China

## Abstract

We explored the phylogenomics and methylomics of *NLR* genes in 41 plant species and found that highly duplicated plant *NLR* genes are hyper methylated in non-CG context.

**Supplementary Information:**

The online version contains supplementary material available at 10.1007/s00299-023-03018-5.

## Introduction

Disease resistance (*R*) genes constitute a class of genes that confer resistance against various pathogens. Nucleotide-binding site and leucine-rich repeat (*NLR*) genes, which comprise one of the largest families of *R* genes, can activate hypersensitivity and induce a series of immune responses after recognizing pathogen invasion. Ultimately, this leads to cell death of infected cells, inhibiting the proliferation and spread of pathogens (Wang et al. 2020). *NLR* genes exhibit very rapid evolutionary patterns in response to various evolving pathogens. Based on the different evolutionary rates, *NLR* genes can be divided into two types: type I *NLR* genes have a fast evolutionary rate and high copy number, whereas type II *NLR* genes are highly conserved and low in copy number (Kuang et al. 2004). The transposon coverage of type I *NLR* loci is significantly higher than that of type II *NLR* loci in terms of differentiation (Luo et al. 2012; Lai et al. 2020). However, the reason why the evolutionary pattern of type I and type II *NLR* genes is associated with flanking transposons remains unknown. Plant genomes carry three types of DNA methylation: gene body CG methylation and transposon-related non-CG (CHG, CHH, where H can be A, T, or C) methylation (Zhang et al. 2018). Non-CG methylation is closely associated with transcriptional silencing of transposons. In this study, we analyzed the methylation and transposon distribution around *NLR* genes using published bisulfite sequencing data of 41 plant species. We found significant differences in the non-CG methylation level between type I and type II *NLR* genes, which suggests that DNA methylation might buffer the cost of the expansion and diversification of *NLR* genes in plants.

## Results and discussion

**Fig. 1:**
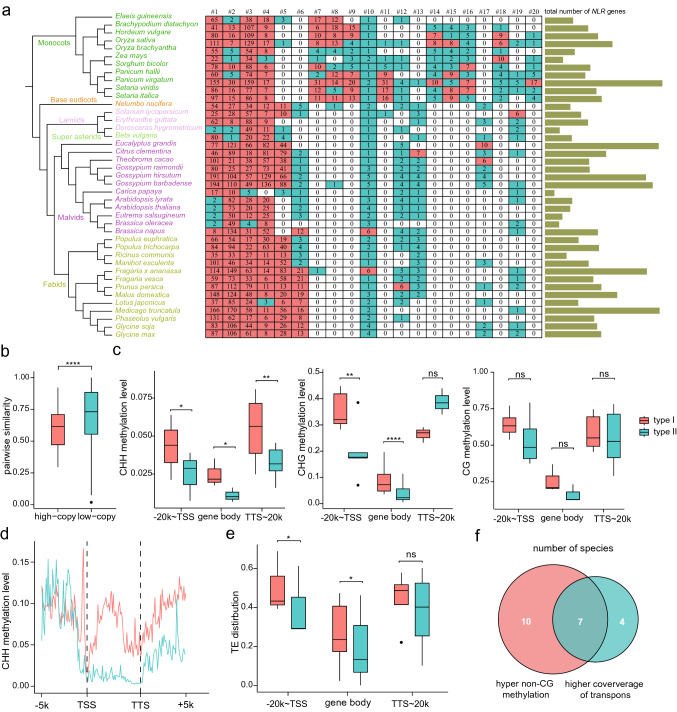
Hyper non-CG Methylation of expanded plant disease resistance *NLR* genes. **a** The number of *NLR* genes in 41 plant genomes (green stripe) and the copy number of per species of the 20 largest *NLR* gene families (decreasing from left to right), with red blocks representing high-copy *NLRs* (> 5), the blue block representing low-copy *NLRs* (≤ 5). **b** The boxplot shows the sequence similarity of high-copy and low-copy *NLRs*. **c** The boxplots show the average CHH, CHG, CG methylation levels of the gene body, upstream 20 kb and downstream 20 kb regions of all type I and type II *NLR* genes in species exhibiting significant differences in methylation levels between type I and type II *NLRs*. Red blocks represent type I *NLR* genes, and the blue block represents type II *NLR* genes (hereinafter the same). TSS denotes transcription start site, and TTS denotes the transcription termination site. **d** CHH methylation levels of type I and type II *NLR* genes in the gene body and flanking 5 kb regions of *Populus trichocarpa*. Methylation levels in the gene body and flanking regions were determined by dividing the region into 100 bins by R package ViewBS and assessed at weighted methylation levels. **e** Boxplots show the transposon distribution in the gene body, upstream and downstream 20 kb regions of all type I and type II *NLR* genes in species exhibiting significant differences in transposon coverage between the two types of genes. Transposon coverage is determined as the percentage of accumulated transposon length in each 5 kb bin. **f** Effect of transposon on methylation level. The number of regions with significant differences in transposons of type I and type II *NLR* genes accounted for the percentage of significant differences in methylation levels

We have identified total 13,208 NBS-encoding proteins from 41 plant genomes using the HMMER search (Tables S1, S2). These proteins were categorized into 509 orthologous/paralogous groups, which represent 509 *NLR* gene families (Table S3). Our aim was to investigate the evolutionary mechanism of *NLR* genes expansion. Therefore, we counted the copy number of *NLR* genes in each family for each species (Fig. 1a), and defined *NLR* genes with a copy number greater than five in each species as high-copy *NLR*s, and those with less than or equal to five as low-copy *NLRs* (See supplementary method, Table S4). We found that high-copy NLRs dominated in the five largest *NLR* families (high-copy: 99.53%, and low-copy: 0.47%), indicating rapid evolution and high amplification of these *NLR* genes. We calculated the pairwise similarity of high-copy *NLRs* and low-copy *NLRs* for each family and observed that the similarity of high-copy *NLR* genes was significantly lower than that of low-copy *NLR* genes (*p*-value = 1.725e−05) (Fig. 1b and Table S5), which suggested that the copy number of *NLR* genes is positively correlated with evolutionary divergence in a certain family. Furthermore, the sequence difference between high-copy and low-copy *NLR* genes was consistent with the evolutionary pattern of type I and type II *NLR* genes. Therefore, we defined *NLR* genes with high copy number (> 5) as type I *NLR* genes and those with low copy number (≤ 5) as type II *NLR* genes.

Previous reports have suggested that the expression levels of *NLR* genes is regulated by DNA methylation (Deng et al. 2017). To test whether DNA methylation affects the expansion of *NLR* genes, we calculated the CG, CHG, and CHH methylation levels in the upstream regions, gene body, and downstream regions of type I and type II *NLR* genes for each species (Tables S6, S7). Among them, 23 species showed significant differences in the upstream, gene body, or downstream regions. The CG methylation levels of type I and type II *NLR* genes were significantly different in 4, 3, and 8 species in upstream, gene body, and downstream consensus regions, respectively, while the CHG methylation levels were 5, 10, and 2 species respectively, and CHH methylation had 3, 3, and 5 species, respectively. We then singled out these regions that showed significant differences in CG, CHG, and CHH methylation levels, respectively, to explore whether there were significant differences in methylation levels of type I and type II *NLR* genes at the overall level (Table S8). The results showed that there were significant differences in the CHH methylation level of the gene body and flanking consensus regions, especially in the downstream consensus regions. There were also significant differences in the upstream and gene body consensus regions of CHG methylation, but no marked changes were observed in CG methylation (Fig. 1c). As an example, we plotted the CHH methylation levels of the *Populus trichocarpa* across the gene body and the flanking region (Fig. 1d). In summary, type I *NLR* genes had significantly higher non-CG methylation levels compared to type II *NLR* genes.

It is widely recognized that DNA methylation has the function of silencing transposons. We speculated that transposons may play a role in DNA methylation-mediated evolution of *NLR* genes in plants. Thus, we calculated the transposon coverage of type I and type II *NLR* genes in upstream regions, gene body, and downstream regions for each species (Table S9). The results showed that the transposon coverage of type I and type II *NLR* genes was significantly different in 3, 7, and 4 species in upstream, gene body, and downstream consensus regions, respectively. We then picked out these regions that showed significant differences in transposon coverage to explore whether there were significant differences in transposon coverage of type I and type II *NLR* genes at the overall level (Table S10). The results showed that there were significant differences in the transposon coverage in upstream and gene body consensus regions (Fig. 1e). Next, we asked whether the difference in non-CG methylation between type I and type II *NLR* is caused by the insertion of transposons. We examined the species having both significantly different non-CG methylation and transposon distribution between type I and type II *NLR* genes. The results revealed that 7 species with substantial changes in non-CG methylation also had marked differences in transposons (Fig. 1f). Thus, the insertion of transposons can partially explain the elevated non-CG methylation around type I* NLRs.*

Plant *NLR* genes are typically lethal to plant cells when expressed at high levels. Therefore, *NLR* genes typically have weak promoters or down-regulated by small RNAs (Zhang et al. 2016). Non-CG methylation is one of the diverse modes of regulation of *NLR* expression. It can buffer gene dosage imbalance caused by gene replication or avoid the cost of resistance in the absence of a pathogen. Insertion of transposons might be one mechanism that elevates non-CG methylation during the duplication of NLRs. We proposed an evolutionary model for the generation of type I *NLR* genes. A transposon was inserted into the promoter or intron of an *NLR* genes, and the non-CG methylation of the transposon extended to the *NLR* gene, suppressing its gene expression. The silenced or down-regulated *NLR* gene was duplicated into multiple copies with no or weak fitness cost. Finally, the descendant genes were recruited to defense pathways if their new mutations make the plant resistant to new pathogens.

In summary, we investigated the association between DNA methylation and *NLR* genes using the genomes of 41 available land species. We found that the highly duplicated *NLR* genes are usually hyper-non CG methylated. Our findings provide new insights into the evolution of *NLR* genes in the context of DNA methylation.

## Materials and methods

All the data used in this study were retrieved from NCBI. We selected the species that had complete genome, gene annotation, and whole-genome bisulfite sequencing (WGBS) data. To identify NLR protein sequences, we used the HMMER V3 of the Hidden Markov model (HMM) corresponding to the Pfam NBS (NB-ARC) family (PF00931; http://pfam.sanger.ac.uk/) with an *e*-value < 1 × 10^–10^. We clustered the NLR proteins into orthologous/paralogous families using OrthoFinder (Emms and Kelly 2019) with default parameters, and then determined the copy number of the *NLR* genes for each species in each family. We calculated the pairwise similarities of high-copy and low-copy NLR proteins using BLASTP and parsed the results using custom scripts.

The reads obtained from WGBS were aligned to their respective genomes using HISAT-3n with default parameters (Zhang et al. 2021). We selected 41 species with WGBS depth greater than five and methylation mapping rate greater than 60% for subsequent analysis. All possible DNA and RNA transposons were annotated using Extensive de-novo TE Annotator pipeline (Ou et al. 2019) with default parameters. Methylation levels and transposon density (the percentage of accumulated length of transposon in each 5kp bin) were calculated in the gene body and flanking 20kp regions of high and low-copy *NLR* genes for each species using custom perl scripts. Significance analysis was performed using t-tests (*p* < 0.001). The methylation levels in the gene body and flanking regions were determined by dividing the region into 100 bins using the R package ViewBS and assessed at weighted methylation levels.

## Supplementary Information

Below is the link to the electronic supplementary material.

**Table S1**: Genomes used in this study. **Table S2**: 13208 NLR proteins in 41 plant genomes were identified using HMMER V3. **Table S3**: The NLR proteins of 41 plants were clustered and divided into 509 families by OrthoFinder. **Table S4**: We counted the frequency of copy number of NLRs in each family of all species and plotted the distribution of copy number. Then, we calculated the first-order difference of the frequency. We selected the value whose difference slope tends to be stable as the cutoff. **Table S5**: The pairwise similarity of NLR proteins of high and low copy *NLRs* in each family was calculated using blast. **Table S6**: Whole-genome bisulfite sequencing (WGBS) data in this study. Including depth of sequencing and the rate for mapping using hiast-3n. **Table S7**: Methylation levels in the upstream regions, gene body, and downstream regions of *NLR* genes with high and low copy of each species were calculated and *t*-test (*p*-value < 0.01 significant). **Table S8**: Sites with significant methylation levels for type I and type II *NLR* genes. **Table S9**: Transposon coverage in the upstream regions, gene body, and downstream regions of *NLR* genes with high and low copy were calculated for each species and *t*-test (*p*-value < 0.01 significant). **Table S10**: Sites with significant transposon coverage for type I and type II NLR genes. (XLSX 455 KB)

## Data Availability

The data that support the findings of this study are available from the corresponding author upon reasonable request.
